# Atlanta Academy of Medicine

**Published:** 1874-03

**Authors:** 


### Atlanta Academy of Medicine.

JAMES B. BAIRD, M.D., Reporter.

Atlanta, Ga., January 5, 1874.

Dr. John G. Westmoreland, First Vice-President, presiding.

Dr. Baird reported the following case: About the 20th of September, was asked to see Julia W., a widow, æt. about forty-five; youngest child eighteen or twenty years old; suffering from painful menstruation. She was temporarily relieved by anodynes, but several days later, she applied to him, suffering intense pain in her back, and a dragging-down sensation in the abdomen. A vaginal examination revealed the presence of a tumor, about the size of the end of a finger, situated on the posterior lip of the os uteri. About two weeks afterward, this was removed and proved to be a mucous cyst. The os was very much enlarged and congested. There being other growths of apparently the same character, around the os, two applications of a saturated solution of chromic acid were made to the external surface around the os, at intervals of ten days or two weeks. Her courses had been regular up to the time of the operation. At the time they should have appeared, after the operation, they did not come on; she continued to improve up to the second menstrual period. There was no menstrual discharge, but she was seized suddenly with peritonitis, fever, vomiting, rigors, intense pain and tenderness, with considerable swelling in the region of the right ovary, extending to the umbilicus, and covering a circular space of about five inches in diameter. This gradually spread over the entire abdomen. Her pulse became feeble and "thready," and she died of asthenia January 3d; about four weeks after her first attack. For a week before her death she vomited freely, and spat up a fluid resembling muddy coffee.

Autopsy—*Fourteen hours after death:* Stomach distended with the fluid above described, about two quarts of which was evacuated; peritoneal coat of portions of the intestines inflamed; evidence of general peritonitis existed. In the region of the right ovary evidences of intense inflammation were found, both in the peritoneum and adjoining cellular tissue. The left ovary healthy, though atrophied from age. The left Fallopian tube admitted a surgeon's probe. The right ovary and right Fallopian tube inflamed. The probe was arrested about midway of the tube. The duct was either obliterated or greatly contracted in diameter for about one-half inch of its length, as a small probe was passed to the obstruction from both ends of the tube, but then met with resistance that could not be easily overcome. The substance and lining membrane of the uterus appeared healthy; the cervical canal was free. Could the cause of peritonitis have been the escape of an ovum into the peritoneal cavity? The uterus with its appendages was presented for inspection.

Dr. Battey was of the opinion that the initial lesion was inflammation of the right Fallopian tube—(Salpingitis); probably supervened upon the removal of the tumor, or the chromic acid applications.

Dr. Taliaferro was present when the applications of chromic acid were made; it was two weeks after the second and last application before the appearance of peritonitis. Thinks it impossible that the inflammation could have been caused by the chromic acid. If it had been, it would have extended from the point of application to the tube, as it was the lining membrane of the uterus was not inflamed, and the inflammation in the tube ceased before it reached the uterine extremity. Inclines to the belief that it was due to the escape of an ovum into the peritoneal sac.

---

ATLANTA, GA., January 12, 1874.

Dr. W. S. Armstrong, President, presiding.

Dr. Battey reported the following case: Mrs. —; married; æt. forty-five. For years has labored under a chronic uterine malady, probably endo-metritis. Her youngest child is fourteen years of age. She has, what he designates, uterine tetanus. She has had these periodical attacks for ten years. They do not occur monthly, but once, twice or three times a year, the masseter muscle on the right side becomes spasmodically contracted. There is, at these times, some tumefaction and a good deal of pain and tenderness. The jaws become fixed, leaving only space sufficient between the teeth for the passage of liquid food, and small crumbs of bread. Excruciating pain in the muscle is sometimes, though not uniformly, present. The paroxysms last from five to fourteen days. His recollection is that it is always the right masseter muscle; no other muscle affected. Her general condition is good—not anæmic. She does not tolerate quinine. He mentioned that in another patient quinine always acted as a drastic purgative.

*Treatment.*—Morphine, and chloroform liniment topically. She is passing through the climacteric period, and he hopes and believes that when the change of life comes, relief from the tetanic symptoms will be secured. She has been under his care for six years. A letter just received from her husband reports another and a very severe paroxysm.

Dr. W. P. Harden inquired if any spinal irritation existed?

Answer—Yes, more or less.

Dr. John G. Westmoreland remarked that some of the symptoms resembled tetanus. The periodical nature of the affection, the disappearance and return of the spasmodic symptoms, is not characteristic of tetanus. It seems to him to be different from what is generally recognized as tetanus. In tetanus, true, we find spasm of the jaw, as in this case; but the convulsions soon become general and violent, and almost always lead to a fatal termination. Regards this as a reflex impression from some local irritation. The only way to prevent its recurrence is to remove the cause; and he does not think dependence upon the change of life the shortest or surest road to a cure.

Dr. Battey added, as a partial rejoinder to Dr. W's remarks: As far as is known of tetanus, it is a condition of the nervous centers caused by reflex irritation. It affects more especially the pharyngeal muscles and the muscles of the neck. Ordinarily tetanus ends in death. But this case should not be excluded on the ground that other muscles do not become implicated, or that it does not end fatally. Here the cause is continuous, or constantly recurring; hence the repeated attacks. In ordinary cases of traumatic tetanus, if death did not ensue, and the condition of the wound continued the same after recovery, the probability is that another attack would follow. Slight causes may produce modified effects. During the late war saw a soldier who presented symptoms of tetanus from a gun-shot wound; stiffness of the masseter muscle lasted a few hours; believes that stimulants and the proper treatment of the wound warded off an attack of tetanus.

Dr. J. G. Westmoreland stated that he had seen a good many cases of traumatic tetanus, some occurring after the wound had entirely healed; only one case recovered, and that was treated by large doses of calomel and quinine. The same treatment adopted subsequently proved unavailing. Has more confidence, since reading the results of some recent experiments, in the chloroform treatment, than any other.

Dr. Armstrong mentioned a case seen during the war. It occurred in a soldier shot through the foot. He had never seen a case get well, and had no hope in this case. Gave chloroform by inhalation, and laudanum. Kept under the influence of chloroform all night and a part of next day; the dangerous symptoms began to subside and recovery ensued.

Dr. Baird observed that hydrate of chloral might prove an efficient palliative in Dr. Battey's case.

Dr. Connally remarked that he had used hydrate of chloral in a case of traumatic tetanus following amputation of the arm, after a gun-shot injury. The patient finally died, but he is satisfied that his life was prolonged by the remedy.

Dr. Battey thought that the most hopeful prospect of cure for tetanus, at present, in our reach, consists in obtunding nervous sensibility, and support to enable the patient to survive until the storm blows over. In reference to chloral, he believes it is admitted by pharmaceutical chemists to be a very sensative chemical, easily undergoing decomposition. He believes that much of the chloral in the market is not strictly up to the standard, and hence it is not safe. His uniform practice is to administer it in small and repeated doses; ordinary dose seven and a half grains, maximum fifteen. Does not approve of the heroic dose recommended by some; never carries its use beyond forty or sixty grains in the twenty-four hours. If that does not accomplish his object he substitutes other articles or uses them in conjunction with it. It is not only idle but unsafe to give chloral for the relief of severe pain. Strictly speaking, it is not an anodyne, but it is decidedly a hypnotic. Sometimes, if patient suffers great pain, gives one, two or three doses, and then a few whiffs of chloroform which produces sleep, and although the pain may return, it is not ordinarily sufficient to arouse the patient.

Dr. Armstrong used chloral at first in large doses, but soon abandoned the practice and now never gives over ten grains. Regards it, under certain circumstances, a very valuable therapeutical agent. In a case where the patient has already taken large doses of opium without any hypnotic effect, and when he is unwilling to push its administration, he finds that a small dose of chloral produces the desired result. Does not give over fifty grains in a day.

Dr. Jno. M. Boring—When chloral was first introduced to the notice of the profession, used it cautiously and with satisfaction; used it recently in delirium tremens; a stout young man to whom opium, valerian, musk and camphor had been given, without producing sleep. Ninety-six hours had passed since he had slept. Was very wild and sweating profusely. Ordered a mustard bath, cold water to his head, and chloral to be repeated in ten grain doses every hour till desired effect was produced. He was asleep a few minutes after taking the first dose, and speedily recovered. In another similar case, occurring in a woman, it was used with equally satisfactory results.

Dr. Battey said that the taste of chloral was very peculiar, and when swallowed the first impulse was to eject it; if this inclination is resisted for a few moments a secondary quieting effect upon the stomach is obtained. Has administered it with decided benefit, even in gastritis.

Dr. W. F. Westmoreland had heard nothing but praises for chloral. There is a dark side. The journals of the country are filled with "deaths from chloral." Dry gangrene is said to have followed its use.

Dr. Calhoun stated that he had the pleasure of a personal acquaintance with the discoverer of chloral, Professor Liebreich. He, Liebreich, believes the use of the remedy is greatly abused in America—so much so that he remarked to Dr. C., on one occasion, that he almost regretted ever having placed it before the profession. He claims much better effects from small doses. Death, in his experiments on animals, has frequently been produced by large doses. The quantity used by him is from five to ten grains. It was introduced solely for its hypnotic properties. Liebreich has called attention to a condition resembling delirium tremens resulting from its protracted use. Also to the danger of a cumulative effect, which it sometimes has.

Dr. Baird's experience in regard to the remedy, confirmed

Dr. Boring's as just given. Had recently given twenty grains, with thirty grains of bromide of potassium, in a most violent case of delirium tremens; a sleep that continued undisturbed for twelve hours, followed its administration in a very few minutes.

Dr. Calhoun mentioned "as a set off" to this case that he had given the chloral, in large doses for two days, to a patient with delirium tremens without apparent effect, when a small injection of morphine produced sleep.

A report was asked for from Dr. W. F. Westmoreland in reference to the case of the young lady, reported several weeks ago, who suffered from a mammary tumor, with paroxysmal attacks of great pain, treated by electricity. In response he said: The paroxysms had become very much less frequent, occurring now about once a month, and evidently associated with her menstrual functions. The neuralgia is not specially confined to the breast. Near the termination of her last menstrual period she had a violent paroxysm which continued for two days. The catamenial discharge is very scanty.

Adjourned.

---

A. W. CALHOUN, M.D., Reporter.

---

Atlanta, Ga., January 19, 1874.

Dr. John M. Boring, Vice-President, in the chair.

Dr. Flewellen reported case of young man with delirium tremens, in which he gave unusual doses of laudanum without effect, when the hypodermic use of morphia had a rapid and most beneficial effect. Would never think of giving an anodyne by the mouth in delirium tremens.

Dr. W. F. Westmoreland asked for information concerning the term uterine tetanus, used to describe a case reported at a former meeting of the Academy. He does not know what is meant by the expression, and thinks the case to be one of hysteria.

Dr. Logan thought the criticism of Dr. W. eminently fitting, and entirely agreed with Dr. W. in reference to the case reported.

Dr. Westmoreland had seen nervous symptoms resulting from hysteria, and suggested that the case of Dr. Battey can be thus classified.

Dr. J. G. Westmoreland reported case of a woman who had ceased menstruating for fifteen years; had been treated for pain in the back and in region of the liver, and for biliary disorder, but without relief. Concluding that the cause of her troubles was seated in the uterus, on exploration found the os and cervix excoriated, the slightest touch producing hemorrhage. He was now satisfied that the uterine condition was the cause of the conditions complained of, although the uterus had not been previously suspected, either by himself or the patient. Upon uterine treatment the patient made good recovery, with, however, a return of the symptoms upon the suspension of treatment; but again brought in subjection by renewal of local measures. He mentions this case to show the fallacy of the opinion that sympathetic nervous disturbances from the uterus do not occur after the change of life.

Dr. Flewellen asked for the treatment in the case.

Dr. Westmoreland replied, twenty-grain solution nitrate of silver; also, Lugol's solution of iodine, applied every fourth day, and then at longer intervals as the case progressed. The solution was applied to all inflamed parts of the organ.

Dr. W. F. Westmoreland is now treating a woman sixty years old, who has a large abscess, and all the train of nervous sympathy alluded to by Dr. J. G. W., which he attributes to hysteria, and thinks we encounter such symptoms often without any uterine disease.

Dr. J. G. Westmoreland mentioned his case to show that these nervous troubles occur in the aged as well as in the young, and he has often likewise seen them in the male, *e.g.*, one now under treatment for a urethral trouble.

Dr. Boring reported a case of infantile erysipelas in a child three weeks old, where the vulva and neighboring parts were much inflamed. He ordered elm poultices, and on the following day found well developed erysipelas extending down the thighs; used lead water with good effect; is now treating it with calomel, bicarbonate soda, tincture iron and quinia; child bears up well, and is now rapidly improving. In the beginning of the treatment he likewise used arnica, with but little effect.

Dr. J. G. Westmoreland reported case of erysipelas in child three or four months old, commencing upon the head, passing over the body and running out at the toes. He treated it with iron, quinine and opium.

Dr. W. F. Westmoreland had seen a few cases of erysipelas in the city within a few days. They were adults; thinks it is probably somewhat epidemic; sees no reason why it should not attack newly-born infants. He is opposed to local applications, used with a view to checking the spreading of the disease. He tested them thoroughly during the war, and found that they accomplished nothing. Poultices are often of use in alleviating the disease, but thinks the tincture of iodine, used to check its advance, never amounts to anything. The effective treatment must be constitutional.

Dr. Boring was sure of benefit from the local application of an ointment of lard and acetate of lead.

Dr. J. T. Johnson considers the disease to be essentially constitutional; no local treatment avails anything; in spite of all remedies it will run its course.

Dr. De Witt treated many cases during the war, and used simply elm or mush poultices, warm or cold, as the patient elected. In his hands the use of tincture of iodine before the application of the poultices had done much good, which may have resulted from the formation of iodide of starch.

Dr. Du Pre had observed good results from cranberry poultices, or compresses saturated with the cranberry juice. It is cooling to the inflamed surface, and the patient at once expresses his satisfaction with the relief obtained. He was called to case of erysipelas in the scalp a few weeks ago, and with the cranberry poultice he observed marked relief to ensue. Hopes the profession may give the remedy a trial, and test its efficacy.

Adjourned.

---

JAMES B. BAIRD, M.D., Reporter.

---

Atlanta, Ga., January 26, 1874.

Dr. W. S. Armstrong, President, presiding.

Dr. Battey reported a case that he has had under observation for three months. A lady, æt. about thirty; mother of one child, æt. six or eight; no history of subsequent pregnancy. Presented usual symptoms of chronic uterine disorder; endo-metritis; not very marked. Has hypertrophy of the cervix so that it protrudes between the labia, resembling, upon a casual examination, prolapsus; lips patulous, slightly everted; fundus in a normal position; uterine cavity measures three and a half or four inches; no sub-involution. Three months ago he detected a movable tumor in Douglas' cul-de-sac on the left side; on the right side felt what he took to be the right ovary, enlarged; both bodies movable. Two months ago re-examined; found slight enlargement of tumor on left, and, he thought, a slight softening. One month ago examined again, and detected decided enlargement in the same tumor; the other—the one on the right side—remained unchanged since the first examination. Last week another examination was made, when it was ascertained that the tumor continued to increase in size, and so much so that it pressed the other tumor upwards, and the uterus over toward the right side; fluctuation was also felt in it. By conjoined taxis, the fingers of one hand firmly pressed up into the posterior vaginal cul-de-sac, may be felt by the hand placed firmly upon the abdomen, to pass between the tumor and the uterus. When the tumor is pressed upon a sickening sensation is produced, similar to that experienced when the testicles or ovaries are compressed. The tumor is easily pressed upward, and freely movable, entirely independent of the uterus. Entertains scarcely a doubt that it is a free, movable, unattached ovarian tumor. Assuming this to be the case, what shall be done? He estimates its present size to be larger than a hen's egg. During the past thirty days it has notably increased, and is becoming softer; thinks it is taking on a cystic character, and that it will soon appear above the superior strait; it can be felt now, by firm pressure, through the abdominal walls. Some time ago Prof. T. Gaillard Thomas brought to the attention of the profession the superior safety of vaginal to abdominal ovariotomy.

Dr. Sims urges, as death in most fatal cases after ovariotomy is due to septicæmia, that an incision be made through Douglas' cul-de-sac to allow the escape of the bloody serum which collects in this situation, and, by establishing a drain, the absorption of the septic material is avoided. Prof. Thomas advances the same idea when urging the claims of vaginal ovariotomy. An outlet is established at the most dependent part; the tumor is removed before attachments are formed, and while it is still small; the operation through the floor of the pelvis would be safe, attended with little mortality; there is no disturbance of the abdominal contents, or exposure of them to the air. It possesses some of the same advantages that subcutaneous tenotomy does over tenotomy through an open wound; and there is no handling of the viscera. He then read from the *New Orleans Medical and Surgical*

*Jonrnal* an account of a vaginal ovariotomy, by Dr. J. T. Gilmore, of Mobile. He remarked that the case just reported presented great facilities for vaginal ovariotomy now; the tumor will, however, soon be too large for removal in this manner. The temptation to divide the thin septum of unimportant structure, and let the tumor out, is very great.

Dr. Armstrong said that the proposition to operate through Douglas' cul-de-sac impressed him favorably. Was much interested in reading Sims' review of Spencer Wells' operations. Thought his suggestion in regard to a drainage tube through the vaginal wall a good one. Has seen Spencer Wells operate, and some of his cases die from what he called pyæmia. Thought it probable that drainage, as recommended by Sims, would have obviated this fatal complication. Advises an early operation when the diagnosis is positive.

Dr. Logan called attention to the value of veratrum viride in convulsive disorders. Its application in this class of cases was first brought to his notice by Dr. W. A. Greene, of Americus, Georgia. Used it recently in a case of puerperal convulsions, in conjunction, however, with the inhalation of chloroform and the abstraction of blood, and was strongly impressed with its good influence. Gave gtt. vi for first dose, repeated in smaller doses.

Dr. Rauschenberg has had no experience with the remedy in convulsions, but, before the war, was applied to by a young German, æt. about twenty, very stout, heavy-set and florid, for epilepsy; had distressing attacks several times a month; had been treated in Germany without benefit; pulse habitually full, strong and unusually frequent. He put him on veratrum viride tincture, six or eight drops, three times a day; the attacks ceased entirely. He was killed at Pensacola; several years having elapsed with no return of the epilepsy.

Dr. Armstrong reported that he had, within a few days, met with one well marked case of meningitis in the city, that would probably prove fatal.

